# Disparities in severe shigellosis among adults — Foodborne diseases active surveillance network, 2002–2014

**DOI:** 10.1186/s12889-018-5115-4

**Published:** 2018-02-07

**Authors:** Lindsey S. McCrickard, Stacy M. Crim, Sunkyung Kim, Anna Bowen

**Affiliations:** Division of Foodborne, Waterborne and Environmental Diseases, Centers for Disease Control and Prevention, 1600 Clifton Road NE, Atlanta, GA 30333 USA

**Keywords:** Shigellosis, Epidemiology, Adult, MSM

## Abstract

**Background:**

*Shigella* causes approximately 500,000 illnesses, 6000 hospitalizations, and 40 deaths in the United States annually, but incidence and populations at risk for severe shigellosis among adults are unclear. This study describes severe shigellosis among US adults.

**Methods:**

We analyzed Foodborne Diseases Active Surveillance Network data for infections caused by *Shigella* among adults ≥18 years old during 2002–2014. Criteria to define severe shigellosis included hospitalization, bacteremia, or death. We estimated annual incidence of shigellosis per 100,000 among adult populations, and conducted multivariable mixed-effects logistic regression to assess associations between severe shigellosis, demographic factors and *Shigella* species among adults with shigellosis.

**Results:**

Among 9968 shigellosis cases, 2764 (28%) were severe. Restricting to cases due to *S. sonnei* and *S. flexneri,* median annual incidence of severe shigellosis among adults was 0.56 and highest overall incidence was among black males 18–49 years old (1.58). Among adults with shigellosis, odds of severe disease were higher among males than females aged 18–49 years old (OR [95% CI] = 1.32 [1.15–1.52], *p* < 0.001) and among males than females with *S. flexneri* infections (OR [95% CI] =1.39 [1.10–1.75], *p* = 0.005). The odds of severe shigellosis were higher among blacks than whites (OR [95% CI] = 1.36 [1.22–1.52], p < 0.001).

**Conclusions:**

Among adults, men 18–49 years old, particularly blacks, have the highest incidence of severe shigellosis. Among adults with shigellosis, severe shigellosis was associated with being male in age group 18–49 years, with infections caused by *S. flexneri,* and with black race*.* Future research should assess associations between severe shigellosis and sexual practices, antimicrobial resistance, comorbidities, and access to care.

## Background

Shigellosis is a diarrheal illness caused by *Shigella sonnei*, *S. flexneri*, *S. dysenteriae*, or *S. boydii*. Shigellosis causes an estimated 500,000 illnesses, 6000 hospitalizations, and 40 deaths annually in the United States [1, 2]. Globally, an estimated 80–165 million cases of shigellosis occur annually, with the majority of cases occurring in developing countries [3]. The route of disease transmission is fecal-oral and the infectious dose is extremely low; as few as 10 organisms can cause illness [1]. Most persons who are infected with *Shigella* develop diarrhea one or two days after infection. Although symptoms are generally mild, antibiotics are frequently prescribed because they slightly shorten the course of the illness and decrease fecal shedding [1]. Children and women have historically experienced higher rates of shigellosis than men, likely due to close contact between affected children and their female caretakers [4]. For reasons that are unclear, among shigellosis infections reported to the Foodborne Diseases Active Surveillance Network [5] since 2009, incidence has been higher among males than females [6].

Shigellosis can cause severe illness and can lead to complications including septicemia, post-infectious arthritis, persistent episodes of gastroenteritis, hemolytic uremic syndrome, or seizures [1, 7, 8]. The severity of illness varies by *Shigella* species. *S. sonnei* and *S. flexneri,* the two most common causes of shigellosis in the United States, typically cause mild diarrhea; however, both pathogens, particularly *S. flexneri*, can also cause bloody diarrhea and cramps [9]. In contrast, *S. dysenteriae* can cause large outbreaks of severe bloody diarrhea, but U.S. cases are rare and usually associated with international travel [3].

Few studies have assessed populations at risk for severe shigellosis. Although young children were at highest risk of *Shigella* related mortality in studies in Bangladesh [10] and the United States, some data now suggest an association between shigellosis severity and HIV-infection [11, 12]. Additional reports have shown disproportionate levels of shigellosis among men who have sex with men (MSM), particularly those with HIV infection [13, 14], and high levels of antimicrobial resistance in *Shigella* isolates from MSM [15, 16]. However, it remains unclear whether disparities in shigellosis severity exist among adult populations. To help clarify, we reviewed laboratory-confirmed cases of *Shigella* infection reported to the Foodborne Diseases Active Surveillance Network (FoodNet) to define the incidence of severe shigellosis and to explore associations between demographic and clinical characteristics and disease severity among US adults.

## Methods

We included all cases of shigellosis among adults aged 18 years or older reported to FoodNet from 2002 to 2014. FoodNet is a collaborative program among the Centers for Disease Control and Prevention (CDC), 10 state and local public health departments, the United States Department of Agriculture’s Food Safety and Inspection Service (USDA-FSIS) and the Food and Drug Administration (FDA) [17]. FoodNet conducts active, population-based surveillance for laboratory-confirmed infections caused by 9 pathogens transmitted commonly through food, including *Shigella* species [17]. The FoodNet catchment area includes approximately 15% of the US population. FoodNet collects clinical and epidemiologic data, including source of isolate, state of residence, hospitalization, and death. Deaths and hospitalizations occurring within 7 days of specimen collection are attributed to the infection. We defined a case of severe shigellosis as *Shigella* infection including hospitalization, bacteremia (i.e., blood was recorded as source of isolate), or death.

We described demographic characteristics of *Shigella* infections by species and disease severity (severe and non-severe), restricting analysis to those for whom age, race, and sex were known for further analysis. We categorized Asian/Pacific Islander and Native American races as “other race” due to small case numbers. Ethnicity was missing in 13.5% of included cases and ethnicity data incompleteness varied by race (10% for blacks, 14% for whites, 18% for other), year (8% to 20%) and across FoodNet sites (1% to 30%) (all *p* < 0.05). Thus, we did not analyze ethnicity data because of concerns about selection bias. We restricted incidence and modeling analyses to shigellosis caused by either *S. sonnei* and *S. flexneri* infections due to the small number of *S. dysenteriae* and *S. boydii* infections. Shigellosis incidence was similar by age across persons 18–49 years old; thus, cases among persons of these ages were combined into one age group for this analysis. We calculated the median annual reported incidence of shigellosis and severe shigellosis per 100,000 persons in the surveillance area by sex, race (black, white, other), *Shigella* species (*S. flexneri, S. sonnei*), and age group (18–49, ≥50 years) using annual U.S. Census Bureau population data for the surveillance area from 2015.

To further examine the associations between severity of shigellosis, demographics, and *Shigella* species among adults with shigellosis, we used a mixed-effects multivariable logistic regression model, treating disease severity as a binary outcome, and sex, age group, race, *Shigella* species and specimen collection year as independent variables. To account for the unknown factors that cause variation in site-specific disease severity rates across FoodNet sites, a random intercept was added in the model. From our exploratory analysis, the risk difference of severe shigellosis by sex was more apparent in 18–49 year olds than in ≥50 year olds. A similar difference was also apparent by *Shigella* species. To assess these potential interactions, we included two interaction terms, one between sex and age group, and one between sex and *Shigella* species. *P*-values < 0.05 were considered significant. All data analysis was performed using SAS 9.3.

## Results

**Fig. 1:**
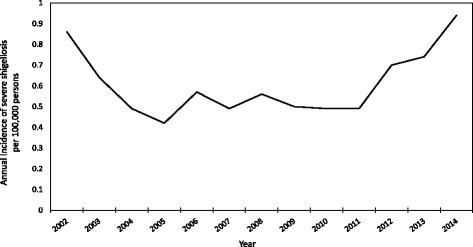
Annual incidence of severe shigellosis per 100,000 persons among adults aged ≥18 years — Foodborne Diseases Active Surveillance Network, 2002–2014

A total of 12,819 shigellosis cases among adults were reported to FoodNet from 2002 to 2014, of which 9968 (78%) had complete age, race, species and sex variables and were used in this analysis. The distributions of race and age were similar among the included and excluded cases, but the included group contained a higher proportion of males than the excluded group (53% vs. 49%). Overall, 7063 (71%) were caused by *S. sonnei*, 2748 (28%) by *S. flexneri*, 111 (1.1%) by *S. boydii*, and 46 (0.5%) by *S. dysenteriae;* 7204 (72%) were classified as non-severe and 2764 (28%) were classified as severe (Table 1). Severe shigellosis was most prevalent among *S. flexneri* infections (34%), followed by those caused by *S. sonnei* (25%), *S. boydii* (21%), and *S. dysenteriae* (20%). Among illnesses classified as severe, 98 (3.5%) fulfilled more than one severity criterion (i.e., hospitalized and bacteremic, or bacteremic and died). Of severe cases, 2735 (99%) were hospitalized, 117 (4%) were bacteremic, and 11 (0.4%) died. Hospitalization length was known for 2653 (97%) of hospitalized patients. Median hospitalization length was 3 days [Interquartile range: 2–4 days]. None of the illnesses caused by *S. dysenteriae* or *S. boydii* resulted in death or bacteremia. Males with shigellosis had a higher proportion of severe illnesses compared with women (30% vs. 25%). More cases of shigellosis were reported among adults 18–49 years old (7658) than among adults ≥50 years old (2310). However, among all reported cases, the proportion of adults ≥50 years old with severe shigellosis (34%) was higher than the proportion of adults 18–49 years old with severe shigellosis (26%). By race, the proportion of severe infections was highest among blacks (35%) compared with whites (25%) or other (25%) race groups (Table 1). Among the 10 sites reporting to FoodNet, Tennessee had the highest proportion (33.6%) and Oregon had the lowest proportion (18.7%) of illnesses that were severe. The median annual incidence of reported shigellosis caused by either *S. sonnei* or *S. flexneri* in FoodNet during 2002–2014 was 2.17 (Interquartile range, or IQR: 1.83–2.33) and was higher for *S. sonnei* than for *S. flexneri* (1.57 vs 0.60, respectively) (Table 2). Severe shigellosis increased in incidence from 2011 to 2014 (Fig. 1). The median annual incidence of severe shigellosis was 0.39 (IQR: 0.31–0.43) for *S. sonnei*, 0.19 (IQR: 0.16–0.23) for *S. flexneri* and 0.56 (IQR: 0.49–0.70) for the two species combined (Table 2). The median annual incidence of shigellosis was 2.22 in males and 1.89 in females, while the median annual incidence of severe shigellosis was 0.63 in males and 0.50 in females. The incidence of both shigellosis and severe shigellosis was higher among persons 18–49 years than adults ≥50 years old, and among blacks than whites.

**Table 1:** Demographic characteristics of shigellosis cases by species of infection and disease severity among adults ≥18 years old— FoodNet, 2002–2014

	All				*Shigella sonnei*				*Shigella flexneri*				*Shigella boydii*				*Shigella dysenteriae*			
	(*N* = 9968)				(*N* = 7063)				(*N* = 2748)				(*N* = 111)				(*N* = 46)			
	Non-severe		Severe		Non-severe		Severe		Non-severe		Severe		Non-severe		Severe		Non-severe		Severe	
	n	(%)	n	(%)	n	(%)	n	(%)	n	(%)	n	(%)	n	(%)	n	(%)	n	(%)	n	(%)
All	7204	(72)	2764	(28)	5271	(76)	1792	(25)	1808	(66)	940	(34)	88	(79)	23	(21)	37	(80)	9	(20)
Sex																				
Male	3706	(70)	1576	(30)	2219	(75)	750	(25)	1434	(64)	810	(36)	41	(75)	14	(25)	12	(86)	2	(14)
Female	3498	(75)	1188	(25)	3052	(75)	1042	(25)	374	(74)	130	(26)	47	(84)	9	(16)	25	(78)	7	(22)
Age (years)																				
18–49	5680	(74)	1978	(26)	4140	(77)	1237	(23)	1456	(67)	727	(33)	59	(86)	10	(14)	25	(86)	4	(14)
≥ 50	1524	(66)	786	(34)	1131	(67)	555	(33)	352	(62)	213	(38)	29	(69)	13	(31)	12	(71)	5	(29)
Race																				
Black	1852	(65)	1004	(35)	1212	(69)	535	(31)	614	(57)	460	(43)	18	(75)	6	(25)	8	(73)	3	(27)
White	4893	(75)	1610	(25)	3756	(76)	1172	(24)	1051	(71)	421	(29)	61	(82)	13	(18)	25	(86)	4	(14)
Other	459	(75)	150	(25)	303	(78)	85	(22)	143	(71)	59	(29)	9	(69)	4	(31)	4	(67)	2	(33)

**Table 2:** Median annual incidence of infections caused by *S. sonnei* or *S. flexneri* by illness severity among adults aged ≥18 years — Foodborne Diseases Active Surveillance Network, 2002–2014

	Incidence per 100,000 (IQR^a^)	
	Severe shigellosis	Shigellosis
All	0.56 (0.49–0.70)	2.17 (1.83–2.33)
Sex		
Male	0.63 (0.53–0.89)	2.22 (1.98–2.75)
Female	0.50 (0.41–0.59)	1.89 (1.78–2.28)
Age group, years		
18–49	0.67 (0.60–0.88)	2.77 (2.27–3.06)
≥ 50	0.39 (0.33–0.45)	1.22 (0.96–1.34)
Race		
Black	1.42 (1.13–1.91)	3.58 (3.45–4.95)
White	0.44 (0.37–0.51)	1.80 (1.66–2.09)
Other	Not assessed^b^	Not assessed^b^
Species		
*S. flexneri*	0.19 (0.16–0.23)	0.60 (0.56–0.64)
*S. sonnei*	0.39 (0.31–0.43)	1.57 (1.24–1.73)

^a^Interquartile range; ^b^Unable to assess because of limited sample size

**Fig. 2:**
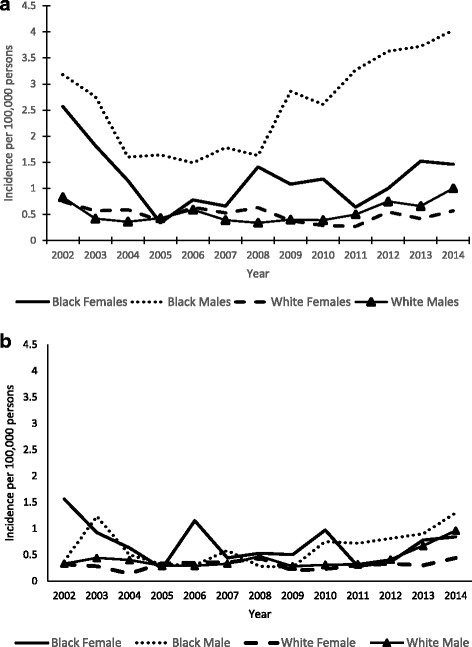
Annual incidence of severe shigellosis per 100,000 persons by race and sex in adults (**a**) 18–49 years of age and (**b**) ≥50 years of age — Foodborne Diseases Active Surveillance Network, 2002–2014

When calculating the incidence of shigellosis by race (black vs white), sex, and age group combined (8 groups in total); black males 18–49 years old had the highest median annual incidence of severe shigellosis (2.76), whereas white females ≥50 years old had the lowest (0.32) (Fig. 2a and b). Among infections caused by *S. flexneri*, black males 18–49 years old had the highest median annual incidence of severe shigellosis (1.58). Among infections caused by *S. sonnei*, black females 18–49 years old had the highest median annual incidence of severe shigellosis (1.08) (data not shown).

In multivariable logistic regression, we observed significant interactions between sex and age group and between sex and species (all *p* < 0.01) among adults with shigellosis. Thus, the associations between severity of infections and sex were presented by age group and *Shigella* species (Table 3). Among 18–49 year olds, the odds of severe shigellosis were higher for males than for females (OR: 1.32, 95% confidence interval (CI): 1.15–1.52, *p* < 0.01). However, among adults ≥50 years old, no significant difference by sex was observed (OR: 1.07, 95% CI: 0.88–1.30, *p* = 0.51). The odds of severe disease were higher for males than females among infections caused by *S. flexneri*, (OR: 1.39, p < 0.01) but not among infections caused by *S. sonnei* (OR: 1.02, 95% CI: 0.90–1.15, *p* = 0.78). Blacks had higher odds of severe disease compared with whites (OR: 1.36, 95% CI: 1.22–1.52, *p* < 0.01) and other race groups (OR: 1.27, 95% CI: 1.02–1.58, *p* = 0.04). The odds of severe shigellosis gradually increased by year during 2002–2014 (OR: 1.04, 95% CI: 1.03–1.05, *p* < 0.01). Site-level random effects were significant (*p* < 0.001), implying significant contextual influences on disease severity (data not shown).

**Table 3:** Factors associated with severe illness among adults aged ≥18 years with shigellosis — Foodborne Diseases Active Surveillance Network, 2002–2014

		Odds Ratio^a^ (95% CI)	*P*-value
Age group, years			
18–49	Male vs Female	1.32 (1.15–1.52)	< 0.001
≥ 50	Male vs Female	1.07 (0.88–1.30)	0.51
Species			
*S. flexneri*	Male vs Female	1.39 (1.10–1.75)	0.005
*S. sonnei*	Male vs Female	1.02 (0.90–1.15)	0.78
Race			
Black vs White		1.36 (1.22–1.52)	< 0.001
Black vs Other		1.27 (1.02–1.58)	0.04
Specimen collection year		1.04 (1.03–1.05)	< 0.001

^a^Results from a multivariable mixed-effects logistic regression model that included sex, age, species, race, data year and two interaction terms between sex & age and species & sex as covariates

## Discussion

This study outlines the burden of severe illness from shigellosis among US adults, and shows significant differences in the risk of developing severe shigellosis by sex, age, race, and species of infection. Among adults in FoodNet, *S. sonnei* caused the greatest number of severe infections, and black men 18–49 years old had the highest incidence of severe shigellosis compared to other race, age, and sex groups. Disparities by sex and race existed: odds of severe illness were higher among men 18–49 years than among women of the same age, among men compared with women infected with *S. flexneri*, and among blacks compared with whites or other races. Although a higher proportion of cases were severe among adults ≥50 years old than among those 18–49 years old, incidence of severe shigellosis was far lower, and no disparities were found by sex, in the older age group. Our data additionally suggest that the incidence of severe shigellosis in adults has been increasing since 2005, whereas shigellosis incidence overall has shown a general decrease from 2009 to 2013 [18].

Race and ethnicity often correlate with social determinants of health, such as poverty, unemployment, and low educational attainment [19]. Racial and ethnic minorities may receive a different level of care compared with non-minority patients; non-Hispanic blacks consistently have the highest rate of potentially preventable hospitalization compared with other racial/ethnic groups in the United States, and have poor access to healthcare [20, 21]. Disparities exist by race and ethnicity for a wide range of infectious diseases, including shigellosis, syphilis, and HIV [22, 23]. Such disparities in sexually transmitted diseases (STDs) in the United States are especially stark, with incidence of chlamydia infection estimated to be 8 times higher in black men than white men in 2013 [24]. In our study, we also found an increased incidence of shigellosis and severe shigellosis among blacks.

In this analysis, men experienced higher incidence of both shigellosis and severe shigellosis than did women. The reason for the male predominance is unknown and we were unable to assess the impact of comorbidities and sexual practices in our findings. It is possible that the increased incidence could be driven by increases in *Shigella* infections in MSM. Between 1975 and 1985, the median age of U.S. males infected with *S. flexneri* increased from 5 to 26 years of age, likely driven by transmission among MSM [25]. In the UK, the incidence of shigellosis among HIV-infected men increased nearly 7-fold during 2004–2015, while incidence among women remained approximately an order of magnitude lower than that of HIV-infected men [13]. Several outbreaks of shigellosis have been documented among MSM internationally, including in the United States and the UK [11, 26], and MSM are at higher risk for shigellosis and STDs compared with women and exclusively heterosexual men [11, 27]. Shigellosis can spread through sexual practices that increase exposure to feces, such as fisting or direct oral-anal contact [11, 28, 29]. The prevalence of behaviors such as unprotected sex, oral-anal contact, and sex with multiple partners, which increase the risk for contracting shigellosis and other STDs, may be increasing among MSM [30, 31]. In addition, sero-sorting, or seeking out sexual partners of the same HIV status, can lead to increases in unprotected sex and further spread of STDs, including shigellosis [32, 33]. Shigellosis has been significantly associated with HIV infection in some outbreaks, and among 3481 domestically-acquired shigellosis cases among men in the UK occurring between 2004 and 2015, 21% were associated with HIV infection [12, 13].

HIV infection is a risk factor for shigellosis [12], and shigellosis severity may be related to immunosuppression or decreased mucosal immunity with HIV infection [34]. Although data are limited, persons co-infected with HIV and *Shigella* generally have longer duration of bacterial shedding in feces, and may suffer from more severe illness [12, 28]. In South Africa, persons infected with HIV were 4.1 times more likely to die from shigellosis compared with non-infected persons [35]. HIV infection may also be associated with antimicrobial-resistant shigellosis, complicating treatment and increasing the risk for more severe illness [36].

Although we could not assess the direct association between antimicrobial resistance and shigellosis severity, antimicrobial-resistant *Shigella* is classified as a “serious risk” pathogen by CDC [37], and data suggest resistance to the preferred therapeutic antimicrobials for shigellosis, including azithromycin, ciprofloxacin, and ceftriaxone, appears to be increasing in the United States [38–40]. An analysis of shigellosis outbreaks reported to CDC found that *Shigella* strains associated with outbreaks in MSM were significantly more likely to be resistant to the key therapeutic antimicrobials than were other *Shigella* strains [15]. Antimicrobial resistance has been associated with increased severity of other diseases, including salmonellosis [41], and can result in longer hospital stays [42]. With increasing prevalence of antimicrobial resistance, shigellosis may last longer and have more severe outcomes.

Our findings suggest severe shigellosis is increasing, especially among men 18–49 years of age, and could be driven by increases among MSM. Shigellosis questionnaires used during public health investigations should incorporate information regarding sexual practices or orientation and concurrent STDs to better define risk groups and guide interventions for severe shigellosis. Because the increase in severe shigellosis may also be associated with increases in antimicrobial resistance, epidemiologic studies should include antimicrobial resistance, treatment and clinical outcome data to further characterize these associations.

Lack of information about sexual orientation or practices, comorbidities such as HIV, and antimicrobial resistance limited our study. Small numbers of deaths and bacteremia cases prevented meaningful sub-analyses for these outcomes, and detailed information about clinical characteristics such as dysentery were not available. Limiting the dataset to cases with complete demographic variables underestimated incidence and may have introduced selection bias. However, since FoodNet is an active surveillance system, we assume no association between the absence of demographic data and disease severity. The racial disparities we found should be interpreted carefully, as non-severe cases of shigellosis may be underreported in groups with poor access to healthcare, including blacks [21], however incidence of shigellosis and severe shigellosis was highest among blacks, suggesting real disparities in burden and risk. Missing data limited our ability to analyze ethnicity, which has documented associations with shigellosis incidence [43]. Combining Asian, Pacific Islander and Native American races into a single category prevents identification of any disparities among these populations, limiting our ability to address the needs of these minority groups. Finally, findings from this study were based on data from only the 10 FoodNet sites. Data may not be generalizable to the US population due to unknown differences in characteristics between the surveillance population and the US population.

## Conclusions

In conclusion, blacks, particularly men 18–49 years old, experienced higher incidence of severe shigellosis compared to other subgrounds and during *Shigella* infections, they had higher odds of developing severe illness during *Shigella* infections, compared to others. The reasons for this are unclear, but may be associated with disparities in social determinants of health, sexual orientation or behaviors, antimicrobial resistance, comorbidities, or circulation of more pathogenic strains within certain populations. Based on these and previous findings, *Shigella* isolates from all persons requiring antimicrobial treatment should be tested for antimicrobial susceptibility. Effective public health interventions for targeted prevention of shigellosis among blacks and men 18–49 years old could dramatically reduce the burden of severe shigellosis. Counseling MSM about prevention of sexual transmission of shigellosis, including guidance about hygiene, use of barriers during sex, and avoiding sex while having diarrhea, is critical. Further studies are warranted to characterize potential associations between severe shigellosis and MSM, antimicrobial resistance, and comorbidities. Among shigellosis patients, clinicians should consider the increased possibility for severe illness among persons infected with *S. flexneri*, males aged 18–49 years old or anyone aged ≥50 years old, and blacks.

## Abbreviations

CDC: Centers for Disease Control and Prevention
FDA: Food and Drug Administration
FSIS: Food Safety Inspection Service
HIV: Human immunodeficiency virus
IQR: Interquartile Range
MSM: Men who have sex with men
STD: Sexually transmitted diseases
USDA: United States Department of Agriculture
